# Advantages of Understanding the Molecular Mechanisms of Angiogenesis in Various Physiological and Pathological Conditions

**DOI:** 10.3390/ijms24065412

**Published:** 2023-03-12

**Authors:** Adel B. Elmoselhi

**Affiliations:** Basic Medical Sciences Department, College of Medicine, University of Sharjah, Sharjah P.O. Box 27272, United Arab Emirates; amoselhi@sharjah.ac.ae; Tel.: +971-6505-7228

The aim of this Special Issue is to highlight the diverse benefits and approaches to studying angiogenesis in various physiological and pathological conditions, such as damaged tissues, impaired embryonic development, cancer progression, and cardiovascular and chronic inflammatory disorders. Angiogenesis is a complex and tightly controlled process through which new blood vessels are formed from existing ones; it is regulated by a range of molecular and cellular interactions and has been implicated the pathogenesis and pharmacological agents of many disorders [1,2,3].

One of the original articles presented in this issue shows the importance of the PI3K/Akt signaling pathway in angiogenesis. It demonstrates that two human recombinant proteins, Arresten and Canstatin, inhibit the angiogenic behaviors of human umbilical vein endothelial cells (HUVECs) by impeding this pathway. This provides new insights into the mechanisms by which these proteins may function as anti-angiogenic agents, and highlights their potential for targeting the PI3K/Akt pathway in the development of new therapies against angiogenesis-related diseases [4]. Another interesting article in this issue reports the role of glycation in inhibiting angiopoietin-1 signaling activation and angiopoietin-1-induced angiogenesis. This study highlights the potential for post-translational modifications of proteins to play a key role in regulating angiogenesis, and suggests that these modifications may be promising targets for the development of new therapies [5]. Furthermore, another article describes a novel high-content angiogenesis assay that reveals how lacidipine, an L-type calcium channel blocker, induces vascular lumen expansion in vitro. This report provides insights into the potential use of lacidipine as a therapy for conditions that are characterized by vascular dysfunction [6]. In addition, we present our own study, in which we provide evidence of the synergistic anti-angiogenic effect of the combination of VEGFR kinase inhibitors, lenvatinib, and regorafenib, which has therapeutic potential for breast cancer. Our study highlights the importance of combination therapies in treating cancer, reducing side effects, and uncovering new avenues for the development of effective treatments [7]. Another in vitro study observed that of several nuclear receptor ligands and miscellaneous compounds, only dexamethasone gave rise to cluster formation, which indicates EC growth and differentiation, similarly to VEGF-neutralizing compounds [8]. Moreover, this issue features an interesting review article on the molecular mechanisms of the plasminogen-activator plasmin system and the inhibition of angiogenesis through the blocking of serine proteases, and how this could generate therapeutic strategies for regulating tumor growth and neovascularization-related disorders [9]. Another review article highlights emerging anti-inflammatory pharmacotherapy and cell-based therapy for lymphedema, a condition that results from disrupted lymphatic flow, leading to impaired tissue repair and wound healing. This report discusses the potential for new therapies to restore lymphatic function and improve outcomes for patients with lymphedema [10]. The other featured articles focus on specific disease contexts, such as “Angiogenesis in Chronic Inflammatory Skin Disorders”. Angiogenesis is shown to be involved in the pathogenesis of various inflammatory skin diseases, such as atopic dermatitis, rosacea, chronic urticaria, and hidradenitis suppurativa. Of particular note is that VEGF—among other inflammatory biomarkers (such as MMP-3), the majority of which come from mast cells—are highly expressed in these conditions. Interestingly, the inhibition of angiogenesis may therefore be an effective therapeutic approach to treating inflammatory skin disorders [11].

Collectively, the articles in this Special Issue provide a comprehensive overview of the molecular mechanisms of angiogenesis in various diseases and conditions. They highlight the complexity of this process and the potential for targeted therapies to inhibit or promote angiogenesis in specific contexts. The findings presented in these articles could lead to the development of novel therapeutic approaches for a range of diseases and conditions that involve abnormal angiogenesis. However, further studies are warranted to better understand the angiogenesis process, and large clinical trials are required to test specific therapeutic targets.
